# Supplementary material for: Improved Cathepsin Probes for Sensitive Molecular Imaging
Source: Molecules. 2022 Jan 27;27(3):842. doi: 10.3390/molecules27030842 (PMC8838171; doi:10.3390/molecules27030842)
Supplement: Supplementary file 1 [file molecules-27-00842-s001.zip › molecules-1527509-supplementary.pdf]

molecules

Supplementary Materials:

Chemical Synthesis

NH_2-Lys-(Resin)-DMBA-AOMK

Solid phase peptide synthesis of Fmoc-Lys(Boc) bromomethyl ketone, **1**.

The following method was adapted from a previous literature procedure [12]; the reaction was conducted under an inert atmosphere—using tubes that were heated and dried with a septum and a $CaCl_2$ plug to avoid humidity. To a solution of Fmoc-Lys(Boc)-OH (1 gr, 2.13 mmol) in anhydrous THF (tetrahydrofuran; 20 mL) at −10 °C, N-methylmorpholine (2.46 mmol, 270 μL, 1.15 eq.) and isobutyl chloroformate (3.01 mmol, 310 μL, 1.25 eq.) were added sequentially. The solution was stirred for 25 min at −10 °C followed by the addition of excess ethereal diazomethane (~5 eq.) that was generated in situ as described in the Sigma-Aldrich Technical bulletin AL-180; see Scheme S1.

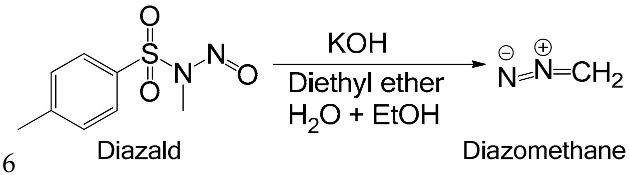

6

Scheme S1. Diazomethane Synthesis. Diazomethane was generated from diazald and KOH as described in [16]. The dissolved diazald (in diethyl ether) was added dropwise through a dropping funnel over 2 h to KOH (dissolved in H_2O + EtOH) that was preheated to 65 °C. The resulting ethereal diazomethane vapors were condensed by dry ice in acetone and were collected in a flask at 0 °C.

The solution was brought to RT over 2 h while stirring. A solution of 22% hydrogen bromide, 44% acetic acid, and 34% DDW (6 mL) was added dropwise, and the resulting solution stirred for an additional 30 s at 0 °C. The reaction was stopped by the addition of ethyl acetate followed by washing of the organic phase with DDW, saturated $NaHCO_3$, and saturated NaCl solution. The final organic layer was dried over $MgSO_4$, and the solvent was removed in vacuo to obtain **1** as a white solid (~20% yield). The product was used without further purification; see **Scheme S2**. MS data: 100% peak of m/z = 445.16 for $[M\text{-}Boc]^+$, 87% peak of m/z = 567.07 for $[MNa]^+$, and 33% peak of m/z = 545.12 for $[MH]^+$. Compound **1** was prepared at least twice.

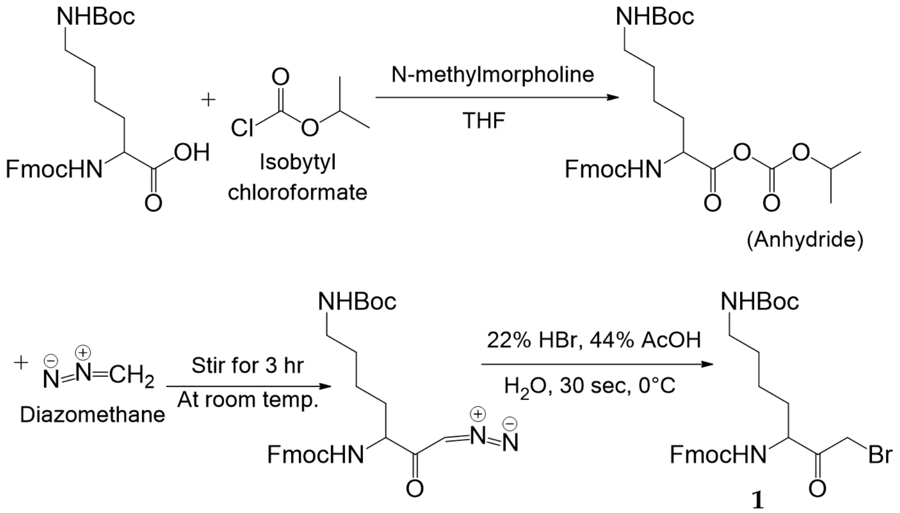

1

Scheme S2. Synthesis of Fmoc-Lysine(Boc) Bromomethyl Ketone **1**. See diazomethane synthesis in **Scheme S1**.

Synthesis of Fmoc-Lys(Boc)-DMBA-AOMK and NH_2-Lys-(Resin)-DMBA-AOMK, **4.**

The crude Fmoc-Lys(Boc) bromomethyl ketone (0.17 mmol, 92.7 mg), potassium fluoride (1.7 mmol 10 eq. 98.8 mg), and 2, 6-dimethyl benzoic acid (DMBA, 5 eq., 0.85 mmol, 128 mg) were dissolved in ~1 mL anhydrous DMF under argon and stirred for 2 h. DCM was added to the crude residue, and the resulting organic phase was washed with DDW, saturated $NaHCO_3$, and brine. The organic phase was dried over $MgSO_4$, and the solvent was removed by rotary evaporation and in vacuo. Crude 2 was purified by C18 reverse phase HPLC using 60–100% DDW-acetonitrile gradient with 0.1%TFA; see Figure 8. Product 2, Fmoc-Lys(Boc)-DMBA-AOMK, was eluted with 74% acetonitrile as a white solid (0.101 mmol, 62.0 mg, 60% yield) that was found to be ~96% pure by LC-MS. MS data: 34% peak of m/z = 615.14 for $[MH]^+$, 100% peak of m/z = 515.22 for $[M\text{-}Boc]^+$, and 55% peak of m/z = 559.10 for $[M{+}Na2\text{-}Boc]^+$. Compound **2** was prepared at least twice.

The Boc protecting group was removed from **2** by incubation with 25%TFA/anhydrous DCM (*v*/*v*) for 20 min followed by DMF and DCM washes. The Fmoc-Lys(NH_2)-DMBA-AOMK, **3** (see structure in Scheme S3), was evaporated and lyophilized for an hour. Purity was found by LC-MS to be ~98%. MS data: 100% peak of m/z = 514.67 for $[MH]^+$ and 29% peak of m/z = 292.16 for $[M\text{-}Fmoc]^+$. 2-Chlorotrityl chloride resin (73.6 mg) was swelled for 35 min in anhydrous DCM and was loaded by shaking with **3**, Fmoc-Lys(NH_2)-DMBA-AOMK (1.4 eq. 62.0 mg 0.101 mmol), and N,N-diisopropylethylamine (DIEA, 3 eq., 38.4 μL, 0.216 mmol) dissolved in anhydrous DCM for 2 h. Methanol (1 mL/gr resin) was added to the resin and shaken for 20 min. Then, the resin was washed twice with DCM and DMF, diethyl ether, dried by vacuum for 10 min, and kept under argon. Resin load was quantified and found to be 0.606 mmol/gr. The resin was swelled in anhydrous DMF for 20 min, then washed with anhydrous DMF, followed by DMF and DCM washes, using anhydrous solvents to obtain **4**, NH_2-Lys-(Resin)-DMBA-AOMK. Compound **4** was prepared at least twice.

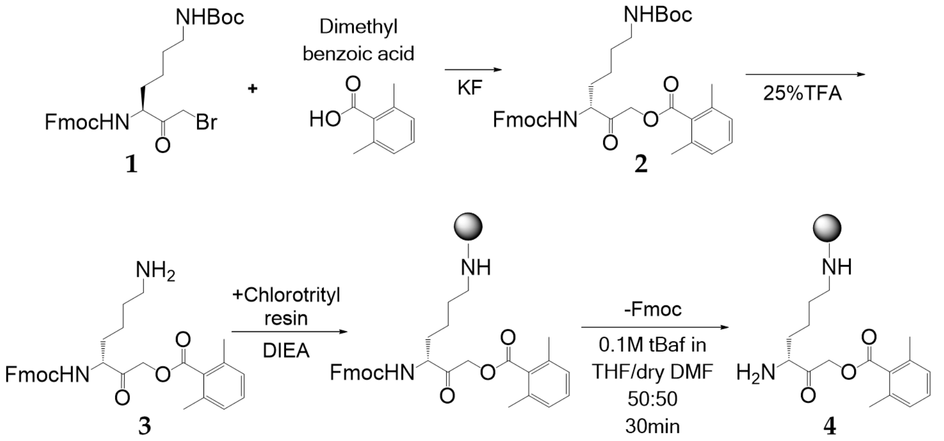

Scheme S3. Synthesis of NH_2-Lys-(Resin)-DMBA-AOMK, **4**.

Synthesis of Aceto-Lys(Boc)-Phe-Phe-COOH and Aceto-Phe-Phe-COOH

Solid Phase Peptide Synthesis of Aceto-Lys(Boc)-Phe-Phe-COOH, **5.**

2-Chlorotrityl chloride resin (500 mg) was loaded by shaking the resin with Fmoc-Phe-OH (3 eq., 1.40 mmol, 540 mg) and N-methyldiisopropylamide (DIEA, 3 eq., 1.40 mmol, 247 μL) dissolved in 5 mL anhydrous DMF for 2 h. Methanol (1 mL/gr resin) was added; the resin was shaken for 20 min and subsequently was washed with DMF and DCM, and dried. Resin loading was found to be 1.2 mmol/gr.

The Fmoc protecting group was removed by incubation with 20% piperidine/DMF (*v*/*v*) for 2 × 15 min, followed by DMF and DCM washes. Then, Fmoc-Phe-OH (3 eq., 1.40 mmol, 540 mg) was coupled by shaking overnight with HOBt (3 eq., 1.40 mmol, 188 mg) and DIC (3 eq., 1.40 mmol, 218 μL) in 5 mL DMF. The resin was washed with DMF and

DCM, and the Fmoc protecting group was removed by incubation with 20% piperidine/DMF (*v*/*v*) for 2 × 15 min, followed by DMF and DCM washes, to form resin bound Phe-Phe-NH_2. Fmoc-Lys(Boc)-OH (3 eq., 1.40 mmol, 654 mg) was coupled by shaking for 2 h with HOBt (3 eq., 1.40 mmol, 188 mg) and DIC (3 eq., 1.40 mmol, 218 µL) in 5 mL DMF. The resin was washed with DMF and DCM; the Fmoc protecting group was removed (as described above), and resin was washed again with DMF and DCM.

The N-terminus was acetylated by shaking for 20 min with acetic anhydride (10 eq., 4.65 mmol, 440 µL) and DIEA (15 eq., 7.00 mmol, 1194 µL) dissolved in 6 mL anhydrous DCM. The resin was dried by vacuum and was washed with DMF and DCM. The Aceto-Lys(Boc)-Phe-Phe-COOH peptide was cleaved from resin by 2% TFA/DCM (*v*/*v*). The cleavage solution was collected, and the solvent was removed by co-evaporation with toluene. The crude peptide was further dried in vacuo to yield **5** as a white solid (362 µmol, 211 mg, 51% yield relative to resin loading) that was found to be ~73% pure by LC-MS and was used without further purification; see Scheme S4a. MS data: 13% peak of *m*/*z* = 583.36 for [MH]+, 100% peak of *m*/*z* = 604.75 for $[MNa]^+$, 27% peak of *m*/*z* = 483.33 for $[M\text{-}Boc]^+$, and 3% peak of *m*/*z* = 505.22 for $[MNa\text{-}Boc]^+$. Compound **5** was prepared at least twice.

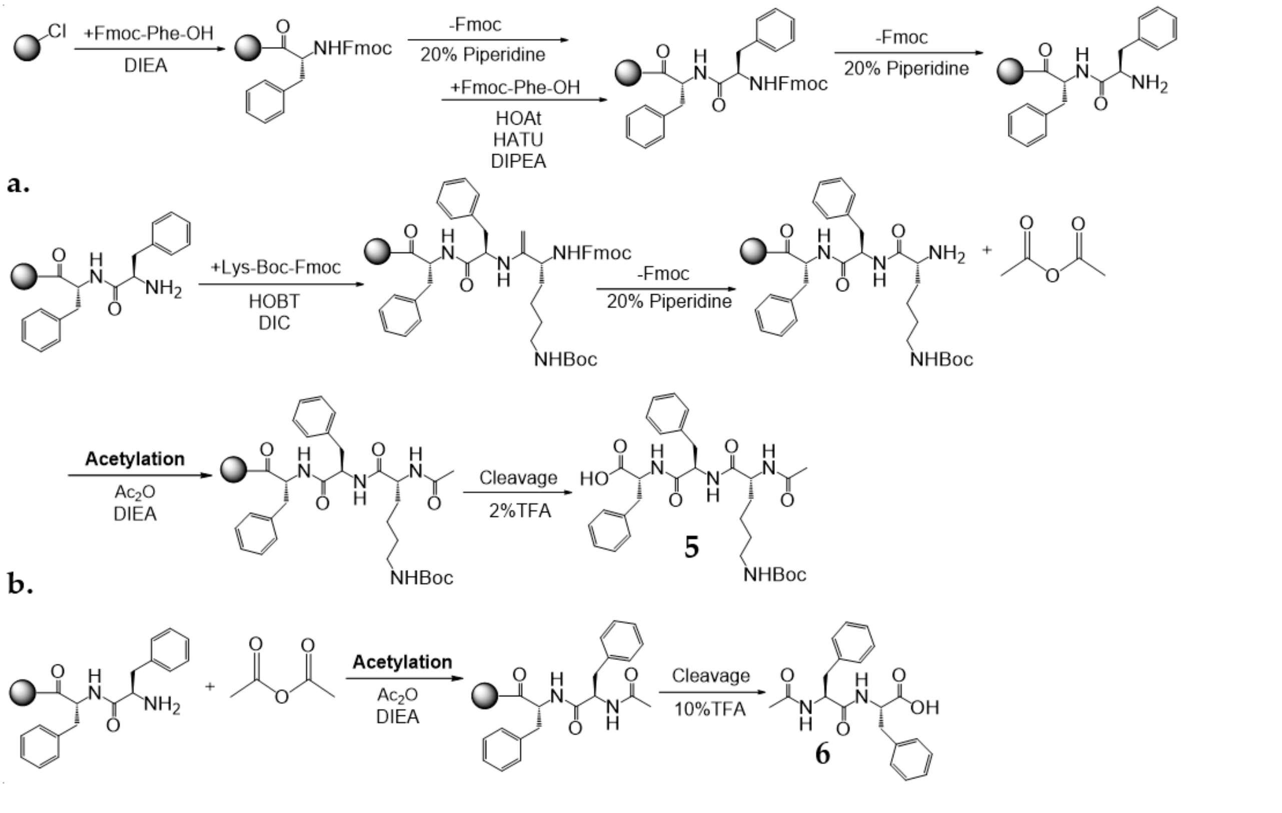

Scheme S4. Solid phase peptide synthesis of a. Aceto-Lys(Boc)-Phe-Phe-COOH **5** and b. Aceto-Phe-Phe-COOH **6**.

Solid Phase Peptide Synthesis of Aceto-Phe-Phe-COOH, **6**.

Next, **6** was synthesized similarly to **5** on a scale that is ~2.5 times smaller; the N-terminus of the Resin-Phe-Phe-NH_2 was acetylated without Fmoc-Lys(Boc)-OH coupling. Moreover, **6** was cleaved with 10% TFA/DCM (*v*/*v*) solution. The cleavage solution was collected, and the solvent was removed by co-evaporation with toluene. The crude peptide was further dried in vacuo to yield **6** as a white solid (246 µmol, 87.0 mg, 94% yield relative to resin loading) that was found to be ~90% pure by LC-MS and was used without further purification; see Scheme S4b. MS data: 100% peak of m/z = 354.92 for $[MH]^+$, 40%

peak of *m*/*z* = 730.75 for [M X2 + Na]$^+$, 20% peak of *m*/*z* = 377.08 for [MNa]$^+$, and 2% peak of *m*/*z* = 708.67 for [MX2]$^+$.

Coupling the two peptides by SPPS and BODIPY TMR-X coupling

Solid Phase Peptide Synthesis of Aceto-Phe-Phe-Lys(NH_2)-DMBA-AOMK, **7**.

The aceto-Phe-Phe-COOH, **6** (3 eq., 79.3 µmol, 28.1 mg), was coupled to the NH_2-Lys-(Resin)-DMBA-AOMK, 4 (1 eq., 26.5 µmol, 13.6 mg), by shaking for 3 h with HOAT (3 eq., 79.3 µmol, 10.8 mg), HATU (3 eq., 79.3 µmol, 30.2 mg), and DIEA (6 eq., 159 µmol, 27.6 µL) in ~3 mL anhydrous DMF (adopted from [17], replacing the NMP by DMF as a solvent). The resin was washed with DMF and DCM, and the final product was cleaved from resin by 10% TFA/anhydrous DCM (*v*/*v*). The cleavage solution was collected, and the solvent was removed by co-evaporation with toluene. Crude **7** was purified by a C4 reverse phase HPLC using 35–37% DDW-acetonitrile gradient with 0.1%TFA; see Scheme S5. The product was eluted with 36% acetonitrile to obtain **7** as a white solid (26.0 µmol, 16.3 mg, 15% yield relative to resin loading) that was found to be ~92% pure by LC-MS. MS data: 100% peak of *m*/*z* = 629.25 for [MH]$^+$ and 5% peak of *m*/*z* = 651.17 for [MNa]$^+$.

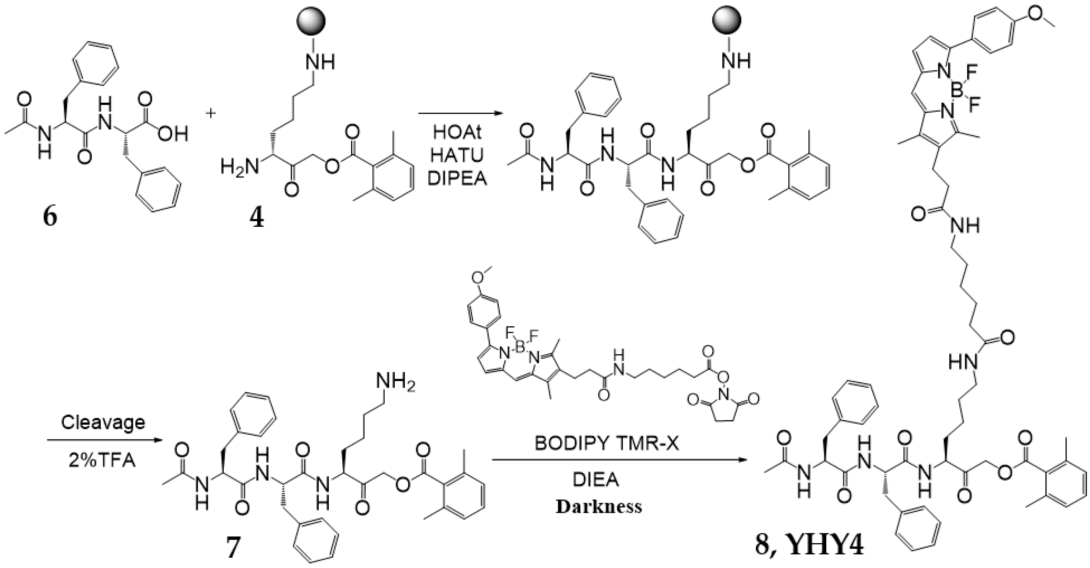

Scheme S5. Synthesis of Aceto-Phe-Phe-Lys(TMR-X)-DMBA-AOMK, **8, YHY4**.

Aceto-Phe-Phe-COOH **6** was coupled to **4** by solid phase peptide synthesis, resulting in peptide **7**, that is cleaved from the resin, followed by coupling to BODIPY TMR-X, resulting in **8, YHY 4**.

Synthesis of Aceto-Phe-Phe-Lys(TMR-X)-DMBA-AOMK **8, YHY4**.

A solution of 0.08 mg/µL BODIPY TMR-X (1 eq., 2.12 µmol, 1.29 mg) in DMSO, compound **7** (1 eq., 2.12 µmol, 1.33 mg), and DIEA (8 eq., 16.9 µmol, 2.9µL) was agitated and allowed to stand in the dark for 2 h. Product **8** was obtained by direct purification from the crude reaction mix by C18 reverse phase HPLC using 55–75% DDW-acetonitrile gradient with 0.1% TFA; see Scheme S5. Compound **8** was eluted with 60% acetonitrile to obtain a pink solid (0.713 µmol, 0.80 mg, 5.2% yield relative to resin loading) that was found to be ~95% pure by LC-MS. MS data: 31% peak of *m*/*z* = 1122.58 for [MH]$^+$ and 100% peak of m/z = 1144.56 for [MNa]$^+$.

Solid Phase Peptide Synthesis of Aceto-Lys(Boc)-Phe-Phe-Lys(NH_2)-DMBA-AOMK, **9**.

The aceto-Lys(Boc)-Phe-Phe-COOH, **5** (3 eq.), was coupled to the NH_2-Lys-(Resin)-DMBA-AOMK, **4** (1 eq.), by shaking for 3 h with HOAt (3 eq.), HATU (3 eq.), and DIEA (6 eq.) in anhydrous DMF (adopted from [17], replacing the NMP by DMF as a solvent).

The resin was washed with DMF and DCM. The resin bound **9** was split; part of it was used to synthesize **10, YHY3**, and pat for **11, YHY2**, and the latter for creating **12, YHY1**; see Scheme S6. The resin bound **9** was prepared at least twice.

Part of the resin bound **9** (53.7 mg) was cleaved from resin by 2% TFA/anhydrous DCM (*v*/*v*). The cleavage solution was collected, and the solvent was removed by co-evaporation with toluene. Crude **9** was purified by C4 reverse phase HPLC using 20–40% DDW-acetonitrile gradient with 0.1%TFA; see Scheme S6a. Product **9** was eluted with 38% acetonitrile to obtain **9** as a white solid (4.03 μmol, 3.45 mg, 19% yield relative to resin loading) that was found to be ~85% pure by LC-MS. MS data: 100% peak of m/z = 857.33 for $[MH]^+$ and 2% peak of m/z = 879.17 for $[MNa]^+$. Compound **9** was prepared at least twice.

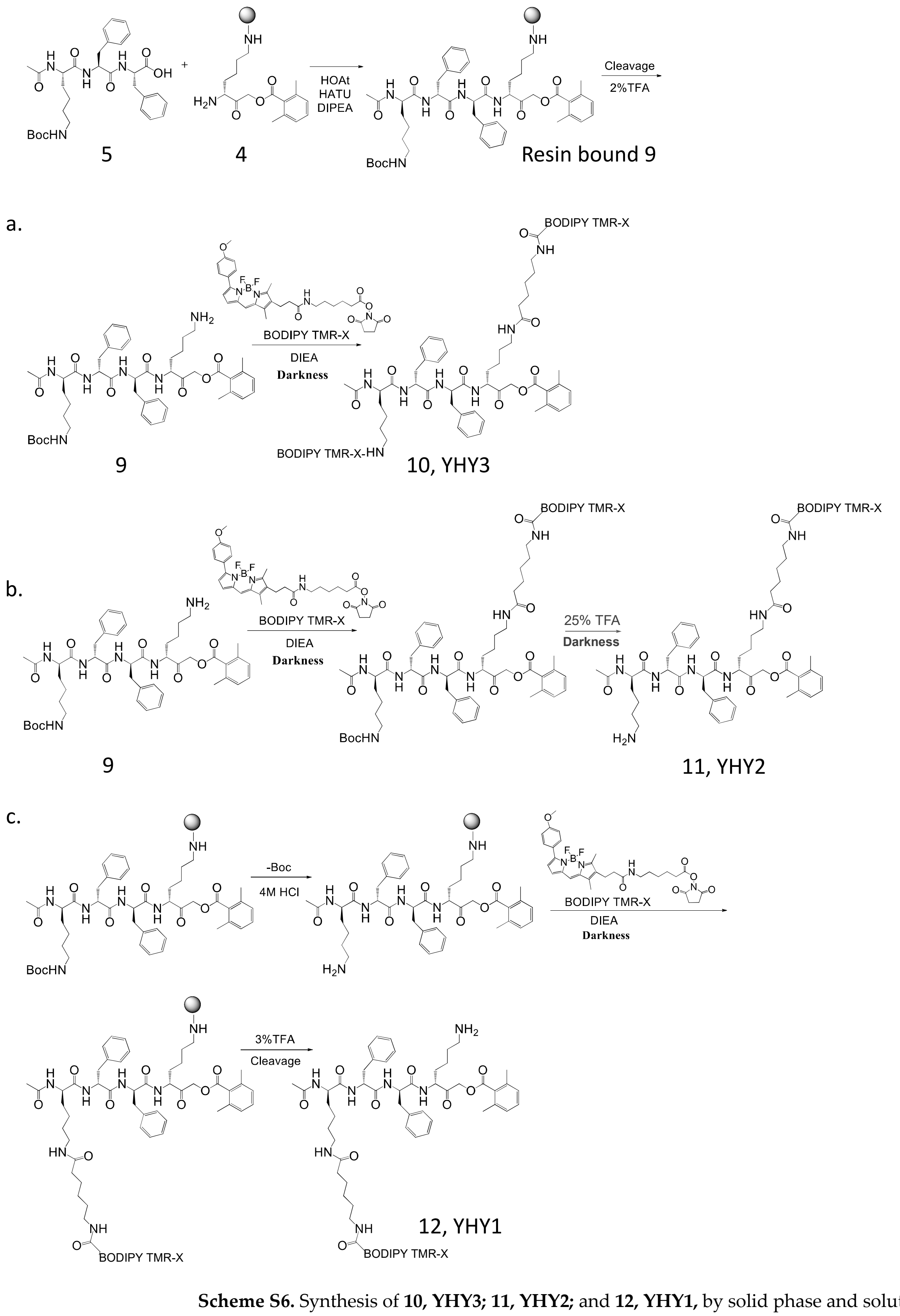

Scheme S6. Synthesis of **10, YHY3; 11, YHY2;** and **12, YHY1,** by solid phase and solution synthesis. The coupling of **5** to resin bound **4** generated resin bound **9**, the precursor for all three compounds. (**a**) Then, **9** was cleaved from resin, and BODIPY TMRX was attached to P1 lysine; the fluorophore also bound to the Boc deprotected P4 to form **10, YHY3**. (**b**) In a different batch, the Boc was kept on the P4, of **9**; BODIPY TMR-X was attached to the P1, and then the Boc was removed to form **11,**

YHY2. (**c**) Compound **9** was kept on resin; the Boc protecting group was removed from P4 lysine, and the BODIPY-TMRX was attached; then **12, YHY1**, was cleaved from the resin.

Synthesis of Aceto-Lys(TMR-X)-Phe-Phe-Lys(TMR-X)-DMBA-AOMK, **10, YHY3**.

A solution of 0.08 mg/µL BODIPY TMR-X (1.24 eq., 2.17 µmol, 1.32 mg) in DMSO, compound **9** (1 eq., 1.75 µmol, 1.50 mg), and DIEA (8 eq., 14.0 µmol, ~2.4 µL) was agitated and allowed to stand in the dark for 2 h. Product **10**, **YHY3**, was obtained by direct purification from the crude reaction mix, by C4 reverse phase HPLC, using 20–80% DDW-acetonitrile gradient with 0.1% TFA; see Scheme S6a. Product **10** was eluted with 57% acetonitrile to obtain a pink solid (0.326 µmol, 0.55 mg, 1.3% yield relative to resin loading). Analysis revealed that **10, YHY3**, has BODIPY TMR-X coupled to both lysine residues as the BOC protecting group was removed during synthesis. The product was found to be ~96% pure by LC-MS. MS data: 45% peak of *m*/*z* = 1743.50 for [MH]+, 30% peak of *m*/*z* = 1765.75 for $[MNa]^+$, 56% peak of *m*/*z* = 1723.67 for $[M\text{-}F]^+$, and 100% peak of *m*/*z* = 852.08 for $[(M\text{-}2F)/2]^+$.

Synthesis of Aceto-Lys(NH_2)-Phe-Phe-Lys(TMR-X)-DMBA-AOMK, **11, YHY2.**

A solution of 0.08 mg/µL BODIPY TMR-X (1.24 eq., 2.93 µmol, 1.78 mg) in DMSO, **9** (1 eq., 2.36 µmol, 2.02 mg), and DIEA (8 eq., 18.9 µmol, ~3.2 µL) was agitated and allowed to stand in the dark for 2 h. The Boc protecting group was removed by incubation with 25% TFA/dry DCM (*v*/*v*) for 20 min, followed by 4 times co-evaporation with toluene. The crude peptide was further dried in vacuo to yield **11** as a pink solid. Crude **11** was purified by C18 reverse phase HPLC using 35–60% DDW-acetonitrile gradient with 0.1% TFA; see Scheme S6a. Product **11** was eluted with 53% acetonitrile to obtain a pink solid (0.416 µmol, 0.52 mg, 4.5% yield relative to resin loading) that was found to be ~88% pure by LC-MS. MS data: 100% peak of *m*/*z* = 1250.58 for [MH]+ and 6% peak of *m*/*z* = 1272.33 for $[MNa]^+$.

Synthesis of Aceto-Lys(TMR-X)-Phe-Phe-Lys(NH2)-DMBA-AOMK, **12, YHY1.**

The Boc protecting group of the resin bound **9**, aceto-Lys(Boc)-Phe-Phe-Lys(Resin)-DMBA-AOMK, was removed by incubation with 4.0 M HCl in dioxane for 2 × 15 min. [16], followed by DMF and DCM washes. BODIPY TMR-X was coupled to 9.75 mg resin bound 9 (8.22 µmol) by incubation with a solution of 0.08 mg/µL BODIPY TMR-X (1 eq., 8.22 µmol, 5.00 mg) in DMSO and DIEA (8 eq., 65.7 µmol, 11.3 µL) for 2 h in the dark. DMF and DCM washes were followed. Crude was cleaved from resin by 3% TFA/DCM (*v*/*v*) to obtain **12**. The cleavage solution was collected, and the solvent was removed by co-evaporation with toluene. Crude **12** was purified by C18 reverse phase HPLC, using 35–60% DDW-acetonitrile gradient with 0.1% TFA; see Scheme S6b. Product **12** was eluted with 53% acetonitrile to obtain 0.472 µmol 12 received from 8.22 µmol 9 (0.59 mg, 8.0% yield relative to resin loading) as a pink solid that was found to be ~95% pure by LC-MS. MS data: 100% peak of *m*/*z* = 1250.67 for $[MH]^+$ and 5% peak of *m*/*z* = 1272.50 for $[MNa]^+$.

Solid Phase Peptide Synthesis of CBZ-Phe-Lys(NH_2)-DMBA-AOMK 13, GB111-NH_2 [12].

CBZ-Phe-OH (3 eq., 77.6 µmol, 23.2 mg) was coupled to the NH_2-Lys-(Resin)-DMBA-AOMK, 4 (1 eq., 25.9 µmol, 13.3 mg) by shaking for 3 h with HOAt (3 eq., 77.6 µmol, 10.6 mg), HATU (3 eq., 77.6 µmol, 29.5 mg), and DIEA (6 eq., 155 µmol, 27.0 µL) in anhydrous DMF (adopted from [17], replacing the NMP by DMF as a solvent). The resin was washed with DMF and DCM, and the final product was cleaved from resin by 10% TFA/anhydrous DCM (*v*/*v*). The cleavage solution was collected, and the solvent was removed by co-evaporation with toluene. Crude **13** was purified by C18 reverse phase HPLC using 35–52% DDW-acetonitrile gradient with 0.1% TFA; see Scheme S7. The product was eluted with 45% acetonitrile to obtain **13** as a white solid (2.43 µmol, 1.40 mg, 9.4% yield relative to resin loading) that was found to be ~98% pure by LC-MS. MS data: 100% peak of *m*/*z* = 574.17 for $[MH]^+$. Compound **13** was prepared at least twice.

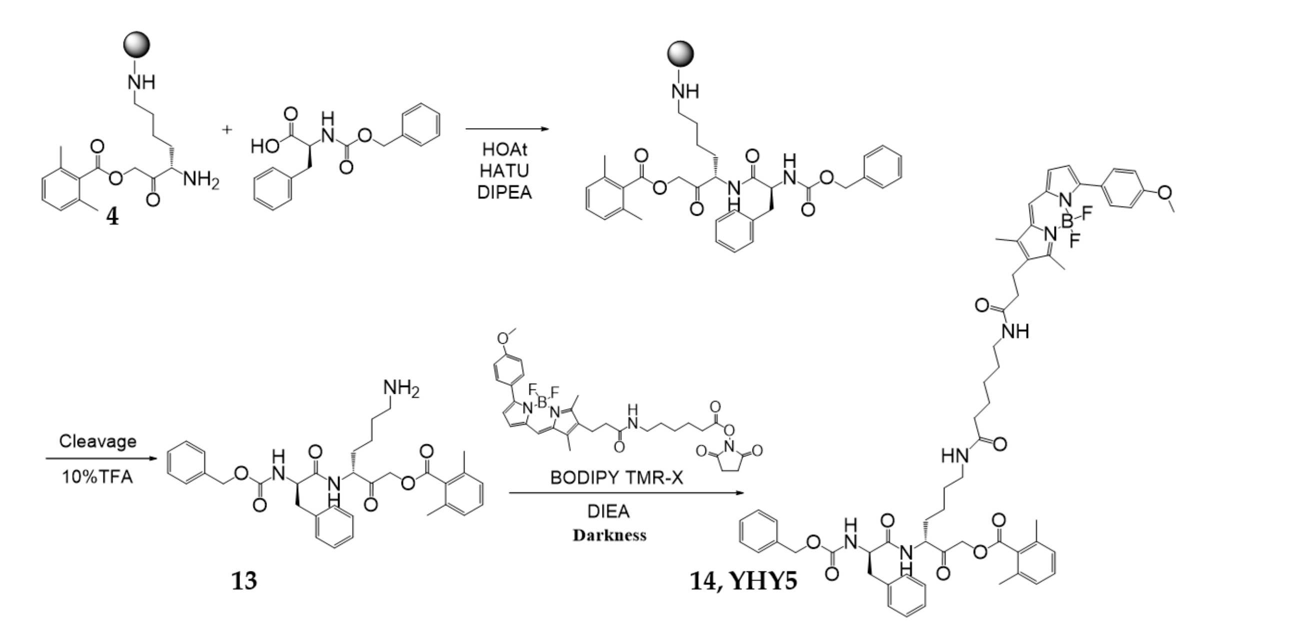

Scheme S7. Synthesis of CBZ-Phe-Lys(TMR-X)-DMBA-AOMK 14, YHY5. CBZ-Phe-OH was coupled to 4 by solid phase peptide synthesis, resulting compound 13 (GB111-NH_2 [12]), that was cleaved from the resin, and BODIPY TMR-X was coupled in solution results in 14, YHY5 (GB111 [12]).

Synthesis of CBZ-Phe-Lys(TMR-X)-DMBA-AOMK **14, YHY5 (GB111 [12]).**

A solution of 0.08 mg/µL BODIPY TMR-X (1 eq., 3.49 µmol, 2.12 mg) in DMSO, **13** (1 eq., 3.49 µmol, 2.00 mg), and DIEA (8 eq., 27.9 µmol, 4.8 µL) was agitated and allowed to stand in the dark for 2 h. Product **14** was obtained by direct purification from the crude reaction mix, by C18 reverse phase HPLC, using 50–81.5% DDW-acetonitrile gradient with 0.1%TFA; see Scheme S7. Product **14** was eluted with 64% acetonitrile to obtain a pink solid (1.63 µmol, 1.74 mg, 4.4% yield relative to resin loading) that was found to be ~95% pure by LC-MS. MS data: 34% peak of 1089.33 for $[MNa]^+$, 5% peak of *m*/*z* = 1067.00 for $[MH]^+$, and 100% peak of *m*/*z* = 1047.42 for $[M\text{-}F]^+$.

Synthesis of 15, tert-butyl (4-((((4-nitrophenoxy)carbonyl)oxy)methyl)phenethyl) carbamate**.** A solution of tert-butyl (4-(hydroxymethyl)phenethyl)carbamate (0.2 mmol, 1 eq.) in 20 mL anhydrous tetrahydrofuran (THF) under argon was mixed with Triethylamine (TEA, 0.6 mmole, 3 eq.), 4-Dimethylaminopyridine (DMAP, 0.01 mmol, 0.05 eq.), and 4-nitrophenyl chloroformate (0.4 mmol, 2 eq.). The reaction mixture was stirred for 2 h at 0 °C, then brought to room temperature (RT) and monitored by LCMS continually for the product; the solvent was evaporated in vacuum, and the product was re-dissolved in DCM. This mixture was washed with brine, dried over Mg_2SO_4, and the solvent was removed in vacuo to obtain **15** as a yellowish solid (0.16 mmol, 82% yield). ESI-MS (*m*/*z*): 317.00 $[M\text{-}Boc+H]^+$; see Scheme S8.

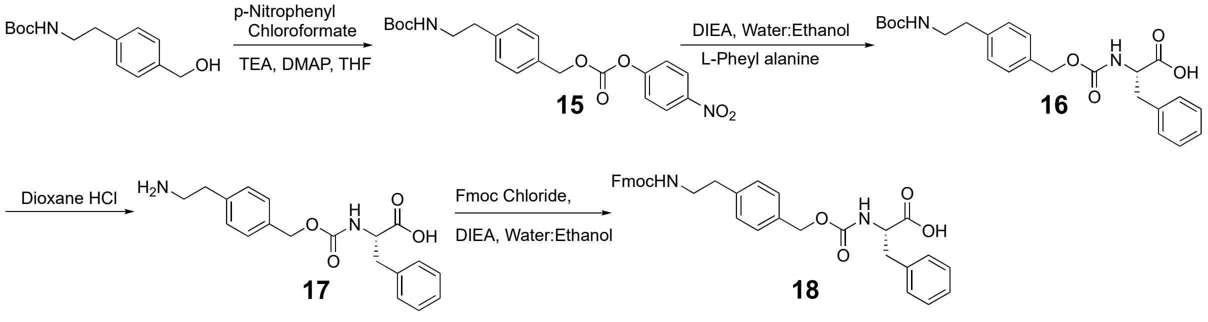

Scheme S8. Synthesis of **18,** (((4-(2-((((9H-fluoren-9-yl)methoxy)carbonyl)amino)ethyl) benzyl) oxy) carbonyl) phenylalanine. Commercial tert-butyl (4-(hydroxymethyl)phenethyl)carbamate was coupled with 4-nitrophenyl chloroformate forming **15** that was coupled to Phe generating **16**. The Boc was removed, and Fmoc was added to protect the free amine forming **18**.

Synthesis of **16**, (((4-(2-((tert-butoxycarbonyl)amino)ethyl)benzyl)oxy)carbonyl)-L-phenylalanine. A solution of Phe (0.12 mmol, 1 eq.) in ethanol:water (1:1, 10 mL) was mixed with DIEA, 0.36 mmol, 3 eq., and **15,** 0.12 mmol, 1 eq. The reaction mixture was stirred for 12 h at an ambient temperature. The solvent was evaporated in vacuum, and the product was re-dissolved in methanol. The crude product was purified by HPLC and eluted with 80% ACN to obtain **15** as a white solid (0.075 mmol, 62% yield), ESI-MS (*m*/*z*): 465.17[M+Na]$^+$, 343.08 [M-Boc+H]$^+$; see Scheme S8.

Synthesis of (((4-(2-aminoethyl)benzyl)oxy)carbonyl)-L-phenylalanine **17**. A solution of (((4-(2-((tert-butoxycarbonyl)amino)ethyl)benzyl)oxy)carbonyl)-L-phenylalanine (3; 0.08 mmol, 1 eq.) in 4N Dioxane HCl (10 mL) was stirred at room temperature for 2 h. The solvent was evaporated in vacuum, and the product was re-dissolved in methanol. The crude product was purified by HPLC and eluted with 45% ACN to obtain **17** as a white solid (0.058 mmol, 72 % yield), ESI-MS (*m*/*z*): 343.08 [M+H]$^+$, 685.00 [M+Na]$^+$; see Scheme S8.

Synthesis of **18,** (((4-(2-((((9H-fluoren-9-yl)methoxy)carbonyl)amino)ethyl)benzyl)oxy) carbonyl) phenylalanine. A solution of (((4-(2-aminoethyl)benzyl)oxy)carbonyl)-L-phenylalanine **(4**, 0.05 mmol, 1 eq.) in ethanol:water (1:1) was mixed with DIEA (0.15 mmol, 3 eq.) and Fluorenylmethyloxycarbonyl chloride (0.1 mmol, 2 eq.). The reaction mixture was stirred for 12 h at an ambient temperature. The solvent was evaporated in vacuum, and the product was re-dissolved in methanol. The crude product was purified by HPLC and eluted with 68% ACN to obtain 18 as a white solid (0.0115 mmol, 23% yield), ESI-MS (*m*/*z*): 565.17 [M+H]$^+$, 587.33 [M+Na]$^+$; see Scheme S8.

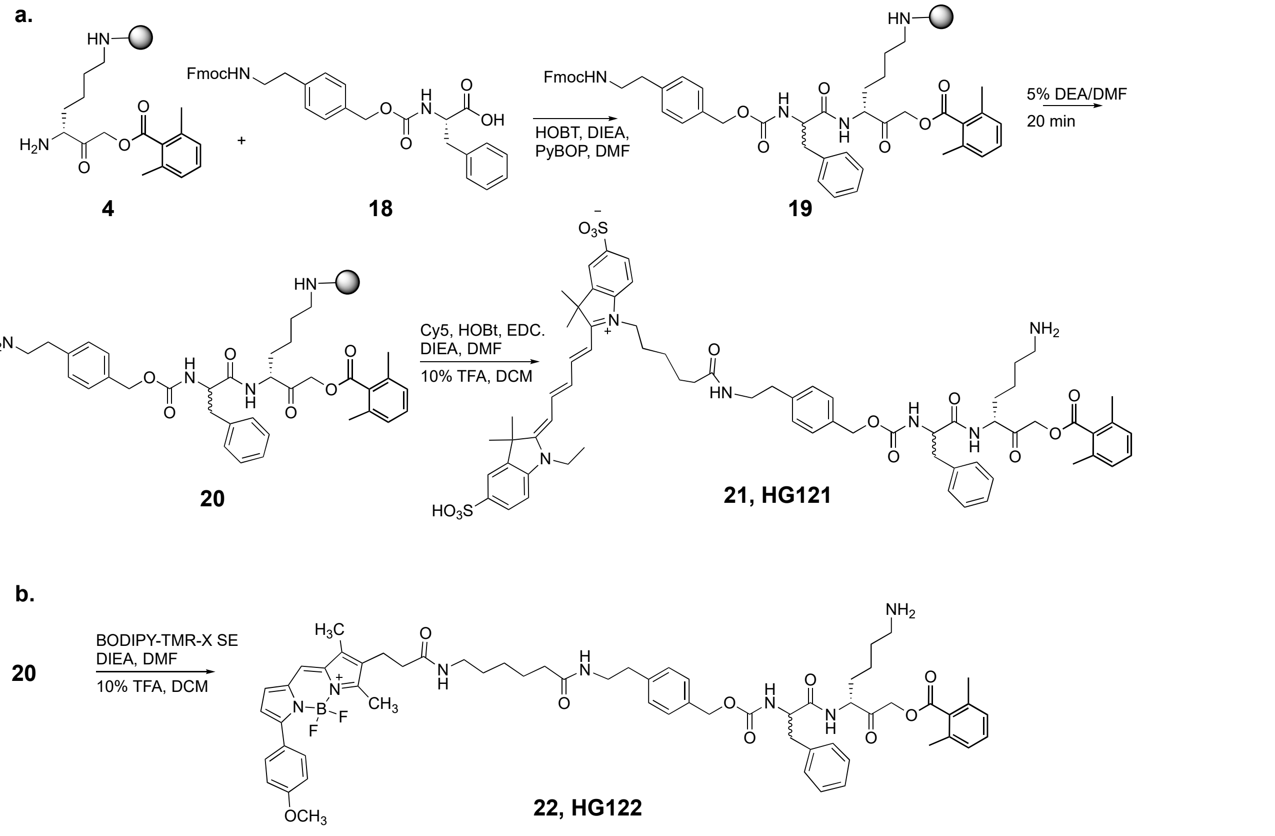

Scheme S9. Synthesis of Cy5 labeled probe **21 (HG121)** and BODIPY TMR-X **22 (HG122).** The Fluorescent compounds were generated by coupling **4** with 19 on resin, removing the Fmoc protecting group, attaching the fluorophore, and cleaving off the resin.

Synthesis of **19**,

The dipeptides resin **19** was synthesized by incubating the resin bound **4** with a solution of Fomc-Ethyl-Cbz-Phenylalanine (**18**, 3 eq.), HOBT (3 eq), PyBOP (3 eq.), and diisopropylethylamine (DIEA; 3 eq) in DMF for 12 h. The resin bound dipeptide **19** was washed with CH_2Cl_2 and DMF.

The Fmoc protecting group was removed from **19** by incubation with 5% Diethyl amine/DMF (*v*/*v*) for 10 min; this step was repeated two times followed by CH_2Cl_2 and DMF washes to obtain the resin bound dipeptide free amine **20**.

Synthesis of **21, HG121**

Compound **21**, **H121** was synthesized by the incubation of resin bound **20** with a solution of sulfo-Cy5-COOH (1.1 eq.), HOBT (3 eq), EDC (3 eq.), and DIEA (3 eq) in DMF for 12 h, similar to in [12,18–20]. The resin was washed with CH_2Cl_2 and DMF. Then, resin was cleaved by the addition of 10% TFA/CH_2Cl_2 (*v*/*v*) for 10 min; this step was repeated six times. The crude product of **21**, **H121**, was purified by preparative HPLC and eluted with 60% ACN to obtain **21, HG121**, as a blue color solid (Yield = ~18%), ESI-MS (*m*/*z*): 1255.58 $[M+H]^+$, 628.42 $[M/2]^+$.

Synthesis of **22, HG122**

Similar to the synthesis of **21**, the BODIPY TMR-X labeled probe, **22**, **H122**, was synthesized by the incubation of resin bound **20** with a solution of BODIPY TMR-X NHS ester (1.1 eq.) and DIEA (3 eq) in DMF for 12 h. The resin was washed with CH_2Cl_2 and DMF. Then, resin was cleaved by the addition of 10% TFA/CH_2Cl_2 (*v*/*v*) for 10 min; this step was repeated six times. The crude product was purified by prep HPLC and eluted with 60% ACN to obtain of **22**, **H122**, as a pink color solid (Yield = ~11%), ESI-MS (*m*/*z*): 1062.00 $[M-48+H]^+$, 1084.00 $[M-48+Na]^+$.
